# Non-alcoholic fatty liver disease (NAFLD): a significant predictor of gestational diabetes mellitus (GDM) and early pregnancy miscarriages—prospective study in Rajarata Pregnancy Cohort (RaPCo)

**DOI:** 10.1136/bmjgast-2021-000831

**Published:** 2022-02-22

**Authors:** Iresha Sandamali Koralegedara, Janith Niwanthaka Warnasekara, Korale Gedara Dayaratne, Farika Nirmani De Silva, Jagath Keerthi Premadasa, Suneth Buddhika Agampodi

**Affiliations:** 1 Department of Anatomy, Faculty of Medicine and Allied Sciences, Rajarata University of Sri Lanka, Saliyapura, North Central, Sri Lanka; 2 Department of Community Medicine, Faculty of Medicine and Allied Sciences, Rajarata University of Sri Lanka, Saliyapura, North Central, Sri Lanka; 3 Maternal and Child Health Research Unit, Faculty of Medicine and Allied Sciences, Rajarata University of Sri Lanka, Saliyapura, North Central, Sri Lanka; 4 Department of Radiology, Teaching Hospital Anuradhapura, Anuradhapura, North Central, Sri Lanka; 5 Department of Obstetrics and Gynaecology, Teaching Hospital Anuradhapura, Anuradhapura, North Central, Sri Lanka

**Keywords:** fatty liver, liver disease in pregnancy, liver imaging, diabetes mellitus

## Abstract

**Background and aims:**

Non-alcoholic fatty liver disease (NAFLD) is increasing globally with a mounting body of evidence on various adverse effects on pregnancy. Yet, prospective studies, especially from low-income and middle-income countries, are lacking in examining the impact of NAFLD in pregnancy. In this study, we explored the effect of NAFLD on the development of gestational diabetes mellitus (GDM) and early pregnancy miscarriages.

**Methods:**

A population-based prospective cohort study was conducted among first-trimester pregnant women who registered in the national pregnancy care programme during July–September 2019 in Anuradhapura district, Sri Lanka. Baseline clinical–biochemical parameters and ultrasound scan (USS) of the liver were done to assess fatty liver. Those who were normoglycaemic based on WHO criteria were followed up, and a repeat oral glucose tolerance test was performed between 24 and 28 weeks of gestation.

**Results:**

Of the 632 pregnant women studied, 90 (14%) and 234 (37%) were diagnosed as having fatty liver grade (FLG) II and I, respectively. The cumulative incidence of GDM in FLG 0, I, and II were 11, 44, and 162 per 1000 pregnancies, respectively. After adjusting for age and other known risk factors, women with FLG II had a relative risk (RR) of 12.5 (95% CI 2.2 to 66.4) for developing GDM compared with FLG 0. In addition, women with FLG I (RR 2.1, 95% CI 1.01 to 4.64) and FLG II (RR 4.5, 95% CI 2.1 to 9.9) were significant risk factors for early pregnancy miscarriages, and FLG II remained as the only independent predictor of miscarriages after adjusting for age, parity, body mass index, blood sugar, blood pressure, and haemoglobin level (adjusted OR 4.2 (95% CI 1.9 to 9.1)).

**Conclusion:**

In this rural south Asian community, NAFLD is shown to be a major risk factor for GDM and early pregnancy miscarriages. Therefore, routine identification of NAFLD through a simple USS may help in the early identification of high-risk mothers.

**Summary box**

**What is already known about this subject?**

- Although there are proven adverse pregnancy outcomes due to non-alcoholic fatty liver disease (NAFLD), it is not routinely recognised in early pregnancy.
- There are no adequate prospective evidence to support the association of NAFLD with gestational diabetes mellitus (GDM) and miscarriages in South Asian community.

**What are the new findings?**

- Fatty liver grade II is shown to be an independent predictor of GDM and early pregnancy miscarriages.

**How might it impact on clinical practice in the foreseeable future?**

- Routine identification of NAFLD through simple non-invasive ultrasound scan may help in early identification of high-risk mothers, hence early interventions to improve maternal morbidity.

## Introduction

Non-alcoholic fatty liver disease (NAFLD) is an initial manifestation of various pathological conditions, such as non-alcoholic steatohepatitis, cirrhosis and liver malignancies.^1^ The most implicated mechanism for NAFLD is insulin resistance.^2^ In the two-hit hypothesis, supra-physiological levels of glucose, sucrose, and fructose can induce lipogenic genes through various mechanisms that lead to de novo lipogenesis and inhibit fatty acid oxidation, causing deposition of fatty acids in various organs such as the liver.^3^ The multiple-hit hypothesis describes several factors, including insulin resistance, hormones secreted by the adipocytes, nutritional factors, gut microbiota and genetic factors responsible for the pathogenesis of NAFLD.

A recent study shows that the prevalence of NAFLD is high in the Asian context due to increasing urbanisation leading to an epidemic of obesity.^4^ The pooled regional incidence of NAFLD in Asian countries is 52 per 1000 person-years compared with 28 per 1000 person-years in the West.^5^ The prevalence of lean NAFLD in Asia is 19%, while it is 7% in the USA.^6^

Although NAFLD is described as the most common liver disease in the West, its effect on pregnancy has not been discussed widely until recently.^7^ Early retrospective studies have reported a low prevalence (28.9/per 100 000 pregnancies) on NAFLD during pregnancy,^8 9^ yet with definitive adverse pregnancy outcomes. Recent studies show a varying degree of NAFLD among pregnant women, with 15% in Canada,^10^ 14.3%–16.7% in the USA,^11 12^ 18.4% in Korea,^13^ and 18.2% in Sri Lanka.^14^ The secondary data analysis of the US inpatient sample of 18 574 225 pregnancies shows that the prevalence of NAFLD after 20 weeks of gestation has tripled over a period of 10 years.^15^ Since its first report in 2011, NAFLD has been identified as a major predictor of many fetal and maternal adverse outcomes, including miscarriages,^16^ gestational diabetes mellitus (GDM),^13 17–19^ hypertensive complications,^14 20^ higher caesarean sections,^14^ intrahepatic cholestasis in pregnancy,^21^ preterm birth,^20^ low birth weight^21^ and postpartum haemorrhage.^8^

Being an insulin-resistant state, pregnancy itself has a higher risk for NAFLD as well as developing hyperglycaemia. Thus, one of the main adverse pregnancy outcomes associated with NAFLD is GDM. The pooled global prevalence of GDM using International Association of Diabetes and Pregnancy Study Group (IADPSG) criteria is reported as 10.6% (95% CI 10.5% to 10.6%),^22^ whereas the estimates for 2005–2015 shows a wide disparity across WHO regions ranging from 5.8% in Europe to 12.9 in the Middle East and North Africa. The incidence of GDM among pregnant women with NAFLD was shown to be more than 20%,^13^ and the severity of NAFLD is proportional to the risk of GDM and large for gestational age babies.^23^ The unconfounded effect of NAFLD on GDM was estimated with an OR around 2 in two prospective cohort studies; one with OR 2.50 (95% CI 1.07 to 5.77)^13^ and another with an OR 2.2 (95% CI 1.1 to 4.3)^10^ and 6.5 (95% CI 2.3 to 18.5) in another study. While early pregnancy NAFLD is almost established as a major predictor of GDM, only a limited number of prospective studies are available in global literature and none from the South Asian region, a region having a high incidence of both NAFLD and GDM. According to our knowledge, none of the prospective studies are available globally about the association between NAFLD and miscarriages.

## Methodology

### Aim

The purpose of the present study was to determine the role of NAFLD as a risk factor for GDM and early pregnancy miscarriages among Sri Lankan pregnant women.

### Study design and setting

This study was carried out as a part of a large population-based prospective cohort study: the Rajarata Pregnancy Cohort (RaPCo). The detailed study design of RaPCo is published elsewhere.^24^ The study was carried out in the Anuradhapura district, the geographically largest district in Sri Lanka. The resident population in the district is 929 539, and in 2019, 15 811 pregnant women registered with the national maternal care programme. Of them, 12 984 were registered in field clinics before eight (8) weeks of gestation, 2063 were registered in field clinic visits at 8–12 weeks of gestation, and 98.6% had at least one clinic visit before delivery.^25^

The minimal sample size to estimate the prevalence of fatty liver was 405. This was based on 95% CI, 0.03 of absolute precision, and a previously reported minimum prevalence of 10%. Data published by Lee *et al* 23 were used to sample size calculation of the cohort study according to the formula published by Kelsey *et al*.^26^ The minimum sample size for cohort study with 95% confidence and 80% power was 543.

### Baseline assessment

**Figure 1:**
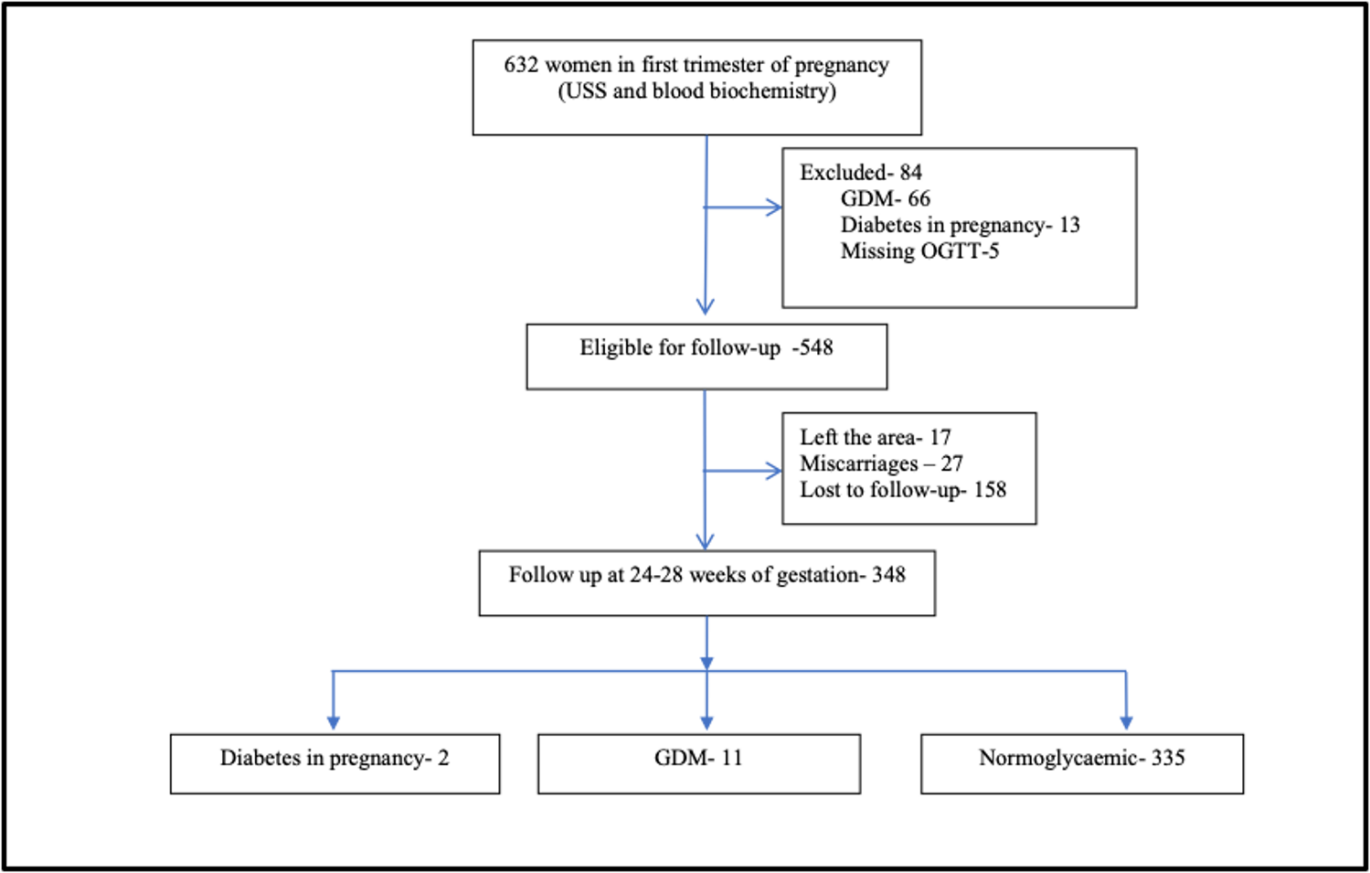
Study flow chart of the participants recruited and loss to follow-up. GDM, gestational diabetes mellitus; OGTT, oral glucose tolerance test; USS, ultrasound scan.

Figure 1 is an overview of the study methodology. Pregnant women with a period of gestation of less than 12 weeks who registered with public health midwives from the end of July to September 2019 were recruited from all medical officers of health areas in Anuradhapura district.

A detailed clinical review, anthropometric measurements, and biochemical tests were performed for baseline assessment. The sociodemographic data collection was done by pretrained medical undergraduates in the third year of training, and MBBS (Batchelor of Medicine and Batchelor of Surgery) qualified medical officers performed clinical data collection and examination. Participants with a history of diabetes mellitus (DM) and currently on treatment for asthma, psychiatric diseases, autoimmune diseases, cardiovascular events, uncertain period of amenorrhea (POA), documented evidence of polycystic ovarian syndrome (PCOS), known liver diseases (except NAFLD), and history of using steatogenic drugs were excluded from this study.

Blood pressure was recorded using a high precision automated blood pressure measuring instrument (Omron Corporation) as the mean of two readings taken 5 min apart from both arms and was categorised as normal and high values for the first and second trimesters according to the National Institute for Health and Care Excellence (NICE) guidelines 2019.^27^

Weight (Wt) was measured using a digital weighing scale, and height was measured using a portable stadiometer. Waist circumference (WC) was measured by placing a non-stretchable fibre-glass measuring tape around the waist midway between the last rib and iliac crest with the subject in the standing position. Hip circumference was measured as the maximum circumference of the buttocks. This entered data was standardised to ensure the routine data were of high quality.^28^ As the weight gain and changes in WC during the first trimester of pregnancy are minimal, we use standard calculation methods and ranges for the Asian adult population to calculate body mass index (BMI), cut-off levels of obesity, and WC pregnancy period.

Blood samples were collected using standard guidelines by qualified nurses. Prerequisites for sample collection were informed to pregnant women prior to the blood collection date. Venipuncture was performed at ante-cubital fossa under aseptic conditions and universal precautions. All these samples were analysed on the same day by an automated analyser (Mindray BS-240 clinical chemistry analyser). Internal quality control was performed before every analytical run. In addition, external quality control was done using peer comparison every month during sample collection and period of analysis of collected blood samples.

An oral glucose tolerance test (OGTT) was performed on all participants at the recruitment and second trimester. Diagnoses of diabetes mellitus in pregnancy (DIP) and GDM were performed using WHO (2016) criteria.^29^ GDM was defined as fasting blood sugar (FBS) of 5.1–6.9 mmol/L and/or second-hour plasma glucose of 8.5–11.1 mmol/L by a 75 g glucose test anytime in pregnancy. Those with FBS ≥7 mmol/L and/or second-hour plasma glucose ≥11.1 mmol/L were labelled as DIP.^29^ In addition, levels of serum aminotransferases, gamma-glutamyl transferases, and serum lipid levels were done as baseline screening tests to identify any liver-related pathological conditions and pre-existing dyslipidaemia.

Ultrasound scan (USS) of the abdomen was performed by competent and qualified investigators using Phillips affinity 70G machine with grey scale, colour Doppler, power Doppler, and spectral Doppler capabilities with curvilinear array transducer in the range of 2–5 MHz. During the procedure, four to five ultrasound images were taken, and the diagnosis of fatty liver was made by a board-certified consultant in radiology on the same monitor under the same lighting conditions with level III ultrasonography evidence to minimise observer bias. At the time of scanning, the radiologist was blinded with patients’ clinical and laboratory data and unaware of previous reports.

Liver echogenicity was compared with the ipsilateral renal cortex and the spleen. In addition, the attenuation of waves, loss of demarcation of the diaphragm, and poor demarcation of the intrahepatic architecture were examined. Thus, fatty liver was graded as follows: Grade 0: normal liver echogenicity; Grade I: diffusely increased hepatic echogenicity but periportal and diaphragmatic echogenicity is still appreciable; Grade II: diffusely increased hepatic echogenicity obscuring periportal echogenicity but diaphragmatic echogenicity is still appreciable; and Grade III: diffusely increased hepatic echogenicity obscuring periportal and diaphragmatic echogenicity.^14^

Although comparison of fatty liver between pregnancy and the general population is not a major objective of the study, a small sample of age-matched randomly selected females was subjected to USS to overcome the possible bias of early pregnancy liver changes.

### Follow-up

All pregnant women with normoglycaemia were followed up, and an OGTT was performed in between 24 and 28 weeks of POA.

### Data analysis

According to the FLG, all continuous variables were summarised as the means with SD. The discrete data were presented as medians. To determine the association between fatty liver grade versus hyperglycaemia and miscarriages, the unadjusted relative risk (RR) was calculated, and to determine independent predictors of outcomes, binary logistic regression was performed, and adjusted ORs were calculated.

## Results

### Baseline assessment

Altogether, 632 pregnant women with POA ≤12 weeks were recruited. The mean age of the sample was 28.5 (SD 5.8) years, and most of them (55.2%) were in the age category of 21–30 years (table 1). The majority (31.5%) of the mothers were in their second pregnancy and had completed post-primary education.

**Table 1:** Baseline characteristics of the 634 first trimester pregnant women recruited for NAFLD assessment

		n (%)
Age (years)	<20	42 (8.8)
	20–24	124 (19.60
	25–29	204 (32.2)
	30–34	160 (25.2)
	34–39	91 (14.4)
	>39	13 (2.1)
Ethnicity	Sinhala	573 (90.4)
	Moor/Malay	53 (8.4)
	Other	891.3)
Gravidity	1	179 (28.5)
	2	197 (31.5)
	3	163 (26.1)
	4 or more	87 (13.9)
Education level	Primary only	9 (1.4)
	Below ordinary level	74 (11.7)
	Ordinary level	360 (56.8)
	Advanced level or more	191 (30.1)
Mother reported diabetes mellitus	Yes	6 (1.0%)
	No	594 (99%)

n, number.

Although the diagnosis of DM was reported by only 6 women, 18 (including the 6 with DM) reported a history of hyperglycaemic detected once or more than once. Prevalence of self-reported hypertension (36, 5.9%), dyslipidaemias (8, 1.3%), hypothyroidism (17, 2.7%) was low. Of the multigravida women, a history of GDM was reported by 17 (2.7%), and the history of having at least one miscarriage was 19.1% (n=121).

Of the pregnant women recruited, 324 (51.2%) had either fatty liver grade I (FLG I) (n=234, 37%, 95% CI 33% to 41%) or fatty liver grade II (FLG II) (90, 14%, 95% CI 12% to 17%). None of the participants had fatty liver grade III (FLG III). The prevalence of FLG I and in the non-pregnant comparison group (n=56) was 38% (n=21) and 14% (n=4), respectively. There was no statistically significant difference between the two groups (χ^2^ 0.006, p=0.997).

All tested liver parameters, biophysical parameters, and OGTT second-hour values gradually increased across the fatty liver grades. The difference between fatty liver grades I and II was higher than the difference between grades 0 and I in all parameters except the FBS value, minimum diastolic blood pressure, portal vein diameter, and dome-to-pole length. FBS in the first trimester was reduced gradually across the FLGs (table 2).

**Table 2:** Comparison of liver parameters, plasma glucose, and biophysical measurements by fatty liver grades among 632 first trimester pregnant women

Variable	Fatty liver grade								
	0 (n=308)			I (n=234)			II (=90)		
	n	Mean	SD	n	Mean	SD	n	Mean	SD
Age (years)	269	27.8	5.7	185	29.4	5.4	62	31.3	5.5
Liver parameters									
Dome-to-pole length (cm)	274	12.7	1.15	198	13.2	1.26	64	13.7	1.14
Portal vein diameter (mm)	263	1.06	0.13	188	1.08	0.14	58	1.09	0.14
AST (U/L)	193	17	5	131	18	6	49	20	8
ALT (U/L)	194	16	7	133	17	10	49	22	12
Gamma GT (U/L)	194	14	7	132	15	9	49	22	13
Blood sugar measurements, mmol/L									
FBS	280	76.8	6.5	203	76.3	6.6	63	76.1	6.8
OGTT second hour	280	103.4	18.3	203	104	18.4	63	110.5	21.3
Biophysical measurements									
Min. systolic BP (mm Hg)	272	101	11	194	103	11	64	107	12
Min. diastolic BP (mm Hg)	272	64	8	194	66	8	64	68	9
BMI (kg·m^-2^)	260	21.9	4.2	188	23.8	4.5	62	27.2	3.9
Waist-to-hip ratio	261	0.82	0.07	181	0.84	0.07	62	0.88	0.06

ALT, alanine aminotransferas; AST, aspartate aminotransferase; BMI, body mass index; FBS, fasting blood sugar; gamma GT, gamma-glutamyl transferase; min. diastolic BP, minimum diastolic blood pressure; min. systolic BP, minimum systolic blood pressure; n, number; OGTT, oral glucose tolerance test.

Of the participants recruited, 84 were excluded from the follow-up study due to detection of DIP (13, 2.1%), GDM (66, 10.4%), and missing OGTT values (5, 0.8%). Of the 548 eligible participants, 17 (3.1%) pregnant women left the area, and from the rest, 27 (5.1%) had late first-trimester or second-trimester miscarriages (figure 1).

Of the rest, 348 pregnant women were assessed at the end of the second trimester. Two cases of DM in pregnancy and 11 GDM cases were detected among previously normoglycaemic women. The incidence of DIP/GDM of FLG 0, I, and II in the second trimester was 11, 44, and 162 per 1000 pregnancies, respectively. The unadjusted RR of developing DIP/GDM in the second trimester compared with FLG 0 was 3.8 (95% CI 0.79 to 19.4) and 12.5 (95% CI 2.6 to 60.0) for FLG I and FLG II, respectively (table 3). The unconfounded effect of FL on developing GDM/DIP was assessed using a binary logistic regression model by adjusting for waist-to-hip ratio, age, parity, FLG, family history of diabetes, female education, and BMI as probable confounders. Only the FLG II has emerged as a significant predictor of DIP/GDM in second pregnancy with an OR of 12.3 (table 3).

**Table 3:** Risk factors for developing GDM/DM in pregnancy

Factor	Adjusted OR	95% CI	Significance (P-value)
Fatty liver grade II	12.3	2.2 to 66.4	0.003
Fatty liver grade I	3.3	0.6 to 18.7	0.166
Age	0.9	0.3 to 2.2	0.829
Parity (primi over multigravida)	2.5	0.6 to 10.2	0.191
BMI	1.4	0.7 to 2.8	0.272
Waist-to-hip ratio	0.8	0.3 to 1.6	0.579
Family history of diabetes	0.6	0.1 to 3.5	0.642
Female education	0.7	0.5 to 1.1	0.170

BMI, body mass index; DM, diabetes mellitus; GDM, gestational diabetes mellitus.

Among 12 pregnant women with GDM (and had BMI assessment), 10 had higher BMI values (sensitivity 83.3%), while 5 had FLG II (sensitivity 41.6%). Out of the five patients with GDM with FLG II, four could be identified by BMI level (>22.9). However, 10 patients with GDM were identified after screening 151 participants with higher BMI (incidence 66.2 per 1000), while 6 mothers with GDM were identified after screening for only 43 participants with FLG II (incidence 139.5 per 1000).

The cumulative incidences of miscarriages among FLG 0, I, and II were 35, 76, and 159 per 1000 pregnancies. Compared with those with FLG 0, pregnant women with FLG I had more than two times the risk (RR 2.1, 95% CI 1.01 to 4.64) of miscarriage. Similarly, FLG II had more than four times the risk of miscarriage (4.5, 95% CI 2.1 to 9.9) compared with FLG 0. The adjusted OR for FLG II compared with both FLG 0 and FLG I was calculated after adjusting for age, parity, blood pressure, blood sugar, haemoglobin, and BMI, and FLG II remained the only independent predictor of miscarriages with a higher level of statistical significance even after adjusting for the above confounding factors (adjusted OR 4.2, 95% CI (1.9 to 9.1).

## Discussion and interpretation

NAFLD during pregnancy partially reflects the physiological changes with fluctuation of oestrogen, lipid levels, and rapid weight gain during pregnancy.^30^ Nevertheless, the link between NAFLD and GDM has been studied in both directions. NAFLD in the first trimester has shown to be a risk factor for dysglycemia in mid-pregnancy,^9^ and having gestational diabetes was also identified as a risk factor for postpartum development of NAFLD.^31^ This study indicates that the prevalence of fatty liver among pregnant women in this rural South Asian community is higher than the reported values from high-income countries (HICs) and the previously reported in the South Asian context. Strengthening the evidence generated elsewhere,^32 33^ this prospective study indicates that NAFLD, specifically FLG II is a major predictor of the development of GDM in pregnancy. Although this association is reported in HICs, ours is the first study to reflect this in the rural Asian population using a community-based prospective cohort study. Even though a previous study reports that NAFLD may not be a significant risk factor for diabetes after adjusting for BMI and age,^9^ our study shows that the FLG II is the only predictor of GDM/DIP, even after adjusting for those confounding variables. The diagnostic thresholds and criteria for GDM are different across the globe and are evolving. The strength of the association in this study is difficult to compare directly with many previous studies due to the differences in GDM diagnostic criteria. De Souza *et al* used similar criteria for GDM and revealed an adjusted OR ranging from 6.8 to 7.8.^32^ Mousa *et al* also reported a significantly higher incidence of GDM among pregnant mothers with fatty liver.^9^ Our estimates are much larger yet have wide confidence intervals (similar to the study by De Souza *et al*).

Although high BMI is a well-recognised risk factor for NAFLD worldwide, the prevalence of NAFLD among low BMI people (lean NAFLD) was high among Asian people than West.^34^ According to our results, the sensitivity of detecting NAFLD is high with a higher BMI (83.3%). However, predictive value of a positive test is very low, making it a poor predictor which was reflected in the logistic regression model. Nevertheless, the number of women with the outcome GDM was inadequate to discuss the higher prevalence of lean NAFLD in this population.

The observed NAFLD prevalence of 51.3% in early pregnancy seems higher than the previously reported values for Sri Lanka (18.2%),^14^ Canada (17.6%),^10^ Korea (18.4%),^13 35^ and the USA (14.3%–16.7%).^12^ However, the prevalence in this study is not significantly different from the comparison group of non-pregnant reproductive-age women from the same geographical location.^36^ Therefore, irrespective of pregnancy, this high prevalence of fatty liver in this rural young women (mean age 28.8 years) needs to be taken seriously as a major predictor of future non-communicable diseases (NCDs).

NAFLD is considered a hepatic manifestation of metabolic syndrome.^37^ Other than GDM, one of the most important findings of this study was the unprecedented observation of FL as a major risk factor for miscarriages with an adjusted OR of 4.2. Liu *et al* showed that abortions in women with NAFLD were higher than those without NAFLD (72.4% vs 69.3%, age-adjusted p=0.001).^16^ The pathophysiology behind this association is still not very clear. However, we can assume that oxidative stress, endothelial damage, and inflammation may predispose to the development of early pregnancy loss.^38^ However, fatty liver is a common finding among PCOS women of reproductive age.^39^ Insulin resistance, growing epidemics of obesity, and androgen excess may contribute to the development of NAFLD among PCOS.^39^ Therefore, the association between fatty liver and miscarriages could be due to PCOS.^40^ Further studies should be planned to determine the association between PCOS, fatty liver, and miscarriages. These observations with a high effect size are of significant public health importance in predicting miscarriages and probably formulating new guidelines for pregnancy care programmes.

Current evidence on diabetes clearly shows that South Asians are at an increased risk of all metabolic derangements, including NAFLD, DM, and GDM.^41^ Combining this knowledge with public health service provisions could be a practical and comprehensive approach to pregnancy care programmes. USS is use for many years as a part of routine pregnancy care, as a ‘dating scan.’ Thus, a simple added step in dating scans to screen for fatty liver during pregnancy may show the risk of developing hyperglycaemia and mid-pregnancy miscarriage. Our multivariable analysis shows that NAFLD is a better predictor of these conditions than the traditional ‘risk factors’ considered in pregnancy. Early identification, monitoring, and intervention for high-risk pregnant women using USS will be a non-invasive screening procedure that could be easily integrated into the system. In addition, the diagnosis of NAFLD in pregnant women would be an early life opportunity to predict and prevent future NCDs among these women.

### Limitations

We used USS to diagnose NAFLD in this study instead of the gold standard liver biopsy. USS is a non-invasive, acceptable, and feasible alternative method, especially in pregnant women. The sensitivity of the diagnosis of fatty liver in USS ranges from 60% to 94%, and the specificity is from 84% to 95%. The sensitivity of detecting fatty liver is increased when the degree of fatty liver increases. In morbid obesity, sensitivity and specificity are reduced from 49% to 75% due to technical errors. However, evidence shows that the reliability of USS for diagnosing fatty liver is higher than that of histology in people with moderate and severe fatty liver. In addition, the grading of fatty liver is subjective and operator-dependent.^14^ The objective of the FL scan in our study is risk prediction rather than a precise diagnosis of steatosis, and for that purpose, routine USS will be adequate.

We used the latest WHO criteria^42^ for diagnosing GDM with an OGTT in the first trimester, which led to the exclusion of 66 pregnant women from the baseline assessment who should not be excluded if the previous classification was used. Since the assessment of glycaemic level is usually done in the second trimester, these pregnant women may not have been excluded in the usual analysis. This exclusion has led to wide confidence intervals showing the need for a larger sample size. However, the estimated risk is the absolute risk of developing hyperglycaemic in the second trimester among those without having any form of hyperglycaemic in the first trimester.

## Conclusion

FLG II, diagnosed by a simple USS, is a major predictor of GDM and early pregnancy miscarriage. The prevalence of NAFLD is higher than that reported elsewhere and in the same context in the past. Therefore, pre-conceptional and/or early pregnancy diagnosis of NAFLD probably incorporated into the routine USS in pregnancy should be considered to identify this important risk factor early. In addition, we recommend incorporating the USS scan to detect fatty liver with the routine dating scan of pregnancy so that additional preventive healthcare could be provided for the mothers having grade 2 fatty liver.

## Data Availability

Anonymous data used in this study will be available from the first author for the researcher on a reasonable request.
